# A Naturally Occurring Bovine Tauopathy Is Geographically Widespread in the UK

**DOI:** 10.1371/journal.pone.0129499

**Published:** 2015-06-19

**Authors:** Martin Jeffrey, Pedro Piccardo, Diane L. Ritchie, James W. Ironside, Alison J. E. Green, Gillian McGovern

**Affiliations:** 1 Animal and Plant Health Agency, Lasswade Veterinary Laboratory, Pentlands Science Park, Bush Loan, Penicuik, Midlothian, Scotland; 2 Laboratory of Bacterial and TSE-agents, Food and Drug Administration, Rockville, Maryland, United States of America; 3 National CJD Research & Surveillance Unit, Centre for Clinical Brain Sciences, School of Clinical Sciences, University of Edinburgh, Western General Hospital, Crewe Road, Edinburgh, United Kingdom; USGS National Wildlife Health Center, UNITED STATES

## Abstract

Many human neurodegenerative diseases are associated with hyperphosphorylation and widespread intra-neuronal and glial associated aggregation of the microtubule associated protein tau. In contrast, animal tauopathies are not reported with only senescent animals showing inconspicuous tau labelling of fine processes albeit significant tau aggregation may occur in some experimental animal disease. Since 1986, an idiopathic neurological condition of adult cattle has been recognised in the UK as a sub-set of cattle slaughtered as suspect bovine spongiform encephalopathy cases. This disorder is characterised by brainstem neuronal chromatolysis and degeneration with variable hippocampal sclerosis and spongiform change. Selected cases of idiopathic brainstem neuronal chromatolysis (IBNC) were identified from archive material and characterised using antibodies specific to several tau hyperphosphorylation sites or different isoforms of the tau microtubule binding region. Labelling was also carried out for alpha synuclein, ubiquitin, TDP43, Aβ_1–42_, Aβ_1–40_. Widespread tau labelling was identified in all IBNC brains examined and with each of seven tau antibodies recognising different hyperphosphorylated sites. Labelling with each antibody was associated with dendrites, neuronal perikarya and glia. Thus IBNC is a sporadic, progressive neurological disease predominantly affecting aged cattle that occurs throughout the UK and is associated with hyperphosphorylation of tau, a rare example of a naturally-occurring tauopathy in a non-primate species. Secondary accumulation of alpha synuclein and ubiquitin was also present. The neuropathology does not precisely correspond with any human tauopathy. The cause of IBNC remains undetermined but environmental factors and exposure to agrochemicals needs to be considered in future aetiological investigations.

## Introduction

Idiopathic brainstem neuronal chromatolysis (IBNC) with variable hippocampal sclerosis is a chronic neurodegenerative disease of adult cattle. It was first reported in 1992 as a distinct pathological subset of clinical bovine spongiform encephalopathy (BSE) suspects occurring in older cows [1]. Between 1988 and 1991 IBNC had a disease incidence in Scotland of 7.16 beef suckers and 2.68 dairy cows per 100,000 cows aged 6 years and over. The youngest recorded case of IBNC is 4 years. The clinical presentation of IBNC, which includes behaviour and locomotor changes with a minority presenting with difficulty in swallowing, are progressive and can be distinguished from other neurological disorders of adult cattle [2,3]. The proximate cause of IBNC cases remains undetermined.

IBNC was originally identified through heightened awareness of adult cattle neurological disease associated with the UK BSE epidemic in the late 1980’s. Cows presenting with clinical neurological disease resembling BSE in this period were examined in the UK under the BSE Orders [4,5]. In the calendar year 1993, at the peak of the BSE epidemic, 27 IBNC cases were recognised amongst BSE suspects in Scotland. Between the years 1988 and 1995 the number of IBNC cases recognised remained at a relatively constant rate of 1–4 per cent of annual confirmed BSE cases and 12–14% of the subset of brains in which BSE was not confirmed (Table 1). The numbers of cases of IBNC recognised amongst BSE suspects fell rapidly as the BSE epidemic declined. As farmers and veterinarians were primed to look for neurological disorders clinically similar to BSE, it is likely that the above numbers underestimate the true prevalence of the disease. With the near-eradication of BSE, few IBNC cases are now recognised annually and aged cows presenting with intractable neurological illness are likely to be killed on economic grounds without post mortem examination of brain tissues. The numbers of IBNC cases now detected amongst clinical BSE suspects in the UK has fallen to less than one per year.

**Table 1:** Table showing annual frequency of IBNC cases relative to negative subset and confirmed cases of BSE in Scotland.

year	No. +ve BSE cases*	No. cases–ve for BSE**	No IBNC cases**	IBNC as % of negative subset	IBNC as % of BSE cases
1988	49	12	0	0	0
1989	208	61	8	13	3.8
1990	496	86	12	14	2.4
1991	808	81	10	12	1.2
1992	1850	112	14	12	0.7
1993***	2208	NA	27	NA	1.1
1994	1326	NA	17	NA	1.2
1995	672	NA	13	NA	1.9

* data from Defra website

** data from personal hand–written notes

*** peak of the UK BSE epidemic

NA data not available.

In the 1990s several pilot studies were undertaken to investigate possible causes of IBNC. In situ hybridisation or immunocytochemical testing for Borna disease virus (two brains), or for Aujeszky’s, Louping-ill or bovine viral diarrhoea virus (five brains) was carried out but antigens to these viruses were not detected (unpublished data). Serum or plasma samples from 5–10 cases of IBNC were examined for levels of Vitamin E, Vitamin B1 and the trace elements and minerals Selenium, Magnesium, Zinc, Iron and Copper. No significant abnormalities were detected (unpublished data). Immunohistochemical labelling for the prion protein (PrP), the protein which accumulates in BSE and other prion diseases, has detected abnormal PrP within the brains of IBNC cases [6]; however, no transmission of a prion like disease has yet been detected in transgenic mice expressing a bovine PrP gene (M Stack personal communication). Immunohistochemical PrP labelling was detected in all 12 IBNC cases examined in this study, but with a staining pattern that is distinct from that of BSE and other more recently recognised atypical cattle prion diseases [6]. This PrP labelling did not mirror the distribution of immunohistochemically detected tau and where frozen tissues were available for testing, protease resistant PrP characteristic of classical forms of prion disease was not detected by Western blotting. Furthermore, we have subsequently found PrP labelling in a minority of diverse neuropathological diseases of sheep [7] and cattle (personal observations), suggesting the presence of abnormal prion protein labelling is not always connected to transmissible prion disease. Although these data are small scale, as yet there is no conclusive evidence to implicate viral, metabolic, trace element deficiency or prion disease in the aetiology of IBNC.

Tau is a microtubule associated protein that occurs predominantly in axons and is central to the assembly of tubulin monomers into microtubules. Tau controls microtubule stability through two key molecular mechanisms. Firstly, an increase in phosphorylation of tau favours de-polymerisation of microtubules [8]; secondly, there are six isoforms of tau formed from alternative splicing, of which three isoforms have three repeat binding domains to microtubules (3R) and 3 isoforms have 4 binding domains (4R). The 4R tau isoforms stabilise microtubules better than the 3R forms [8]. Tau was first implicated as a protein involved in neurodegenerative disease when it was discovered to be a major component of the neurofibrillary tangles found in Alzheimer’s disease patients [9]. Subsequently, neurofibrillary tangles and other lesions composed of aggregated hyperphosphorylated forms of tau proteins (P-tau) were found in a wide range of neurodegenerative conditions, which are collectively known as tauopathies (Table 2) [9,10].

**Table 2:** Table showing characteristics of some human tauopathies compared with IBNC.

Disease	Western blot bands (kDa)			No. repeats	Nature filaments	Other distinguishing features
	60	64	69			
Alzheimer’s disease	+	+	+	3R & 4R	Paired, helical twisted filaments	Widespread Aβ plaques
Pick’s Disease	+	+		3R	Random filaments	Fronto-temporal cortex with absence of Ser 262 & Ser356 sites.
Corticobasal degeneration		+	+	4R		Glial tauopathy with astrocytic plaques
Progressive supranuclear palsy		+	+	4R	Straight filaments	Mainly limbic system including brainstem Glial tauopathy with tufted astrocytes
Argyrophilic grain disease	+*	+*	+*	4R		Usually accompanies other conditions
Parkinson’s disease	+	+	+	3R 4R	Paired helical filaments	Neuronal loss in striatum/substantia nigra. Presence of Lewy bodies
IBNC	NA	NA	NA	3R	No inclusions	Brainstem neuronal degeneration

*variable Western blot immune-phenotypes are reported in Argyrophilic grain disease [28].

Despite the well recognised and wide ranging spectrum of human disorders associated with P-tau, no generalised tauopathy has yet been described in association with discrete neurological deficits in domestic animals. P-tau with neurofibrillary tangles are reported in aged simian species, rabbit, bear, guanaco, reindeer and bison at sites that may correspond to the initial pathology of Alzheimer’s disease [11], but these lesions do not appear to be progressive. Similarly, both intra-cellular tau and glial associated tau are linked to the accumulation of plaques in monkeys over the age of 20 years [12]. Neurofibrillary tangles are also reported in sheep [13] and cattle [14] in the absence of clinical disease. We report here that IBNC affected cows show severe and widespread accumulation of P-tau, ubiquitin and alpha synuclein indicating IBNC to be the first recognised naturally occurring clinical animal tauopathy.

## Materials and Methods

No animal was killed for the purposes of this study. All tissues were obtained from cows suspected of clinical BSE and killed as notifiable disease suspects under the UK government BSE orders (The Bovine Spongiform Encephalopathy Orders (1998) Statutory Instrument No’s 1049 and N0 2299).

Between 1988 and 1995, 101 cases of neuropathologically defined IBNC were recognised amongst brains from clinical BSE suspects submitted to our laboratory. Cases of IBNC have continued to occur up to the present day but the means of reaching diagnosis on BSE suspects has changed making it problematic to be sure of the actual numbers and proportions of IBNC cases submitted relative to the BSE suspects. Formalin fixed- paraffin embedded blocks from confirmed cases of IBNC were retrieved from archive. Twelve cases were selected for further immunohistochemical analysis, representing brains with a range of pathological features, (including those with hippocampal sclerosis or spongiform change and those without). Additional samples of BSE and other unrelated bovine neurological diseases such as encephalic listeriosis, malignant catarrhal fever, and brains from cows aged 5 to 16 years with no histological lesions that were contemporary to the IBNC brains, were also sourced and included as controls.

Frozen tissues from the majority of IBNC brains were not routinely retained. However, following the recognition of P-tau by immunohistochemistry, three IBNC cases with frozen brain samples were obtained from within the resources of the Biological Archive Group at the Animal and Plant Health Agency, Weybridge. Immunohistochemistry using the AT8 antibody confirmed the presence of P-tau in brainstem in all three cases and 3–5g of frozen tissue from cerebellum hippocampus and thalamus was retrieved from them for Western blotting. Additionally frozen brain tissues from two adult cattle with no significant histological lesions and a case of encephalic listeriosis were sought as controls. These controls were AT8 P-tau negative by immunohistochemistry.

For histology and immunohistochemistry tissue sections were cut at 5μm according to standard procedures for light microscopy and stained by Haematoxylin and Eosin (HE) or Bielschowsky silver stain for detection of neurofibrillary tangles. Immunohistochemistry was performed on sections of cerebral cortex, striatum, hippocampus, thalamus, midbrain, cerebellum and brainstem using primary Tau antibody AT8 (Thermo-Scientific, Northumberland, UK), and tau antibodies specific to hyperphosphorylated threonine (T) T205, T212, T231 (Millipore, Watford, UK), and serine (S) S214 (Millipore, Watford, UK), S396, S404 (2BScientific, Upper Heyford, UK) residues and also to RD3 and RD4 (Millipore, Watford, UK) which recognise three (3R) and four repeat (4R) binding domains of the microtubule binding region. Labelling on selected sections was also carried out for: the Alzheimer’s associated proteins Aβ_1–42_ and, Aβ_1–40_ (AbD Serotec, Kidlington, UK); the Parkinson’s disease associated protein alpha synuclein (Milipore, Watford, UK); the hyper-phosphorylated, ubiquitinated and 43 kDa cleaved form of the transactive response (TAR) DNA-binding protein known as pathologic TDP43 (Millipore, Watford, UK), which is a major disease protein of frontotemporal dementia and motor neurone disease; and Ubiquitin (Dako UK Ltd, Ely, UK) which is abnormally accumulated in the presence of aggregated tau, alpha synuclein and other misfolded proteins.

Sections were labelled according to the protocol in routine use in our laboratory [15], with the exception of RD3 and RD4 antibodies which require specific pre-treatments to ensure discrimination from intracytoplasmic expression of native forms and P-tau. Immunolabelling for 3R and 4R tau was carried out using routine procedures and additionally following recommended pre-treatments [16].

Purification of tau was carried out on 1g of frozen grey matter sampled from the hippocampus, thalamus and cerebellum of each of the three confirmed cases of IBNC and each of the control brains using the purification protocol described by Lee *et a*l [17]. The resulting high speed pellets containing tau were re-suspended in 100μl of sample buffer and stored at -80°C for Western blot analysis.

Western blot analysis was carried out on 5μl samples of brain extracts, diluted 1:2 in sample buffer. Samples were denatured by boiling at 100°C for 5 minutes. Polyacrylamide gel electrophoresis and Western blotting was performed using the NuPAGE Novex 10% Bis-Tris gels (1.0mm) gels (Life Technologies, Paisley, UK). Briefly, proteins were separated by running at 40mA for 2 hours in 1X NuPAGE MES running buffer. Proteins were transferred onto polyvinylidene difluoride (PVDF) membrane (Bio-Rad, UK; product 162–0177) at 30V (constant current) for 1 hour using the NuPAGE transfer system. Membranes were probed using the bovine monoclonal antibody anti-tau-1, clone PC1C6 (Millipore, Watford, UK). This antibody is not specific for hyperphosphorylated forms of tau.

Araldite embedded tissues blocks from brainstem or hippocampus were available from 2 cases where tissues had been fixed in mixed aldehydes. These were cut at 60μm, stained with uranyl acetate and lead citrate and examined using a Jeol 1200Ex electron microscope.

## Results

Neurohistologically, IBNC is characterised in brainstem by widespread degeneration of neurons including chromatolysis and Wallerian type degeneration of white matter tracts. These latter are often prominent in radices of cranial nerves. In a proportion of cases extensive neuronal loss occurs in the CA1 and dentate gyrus of the hippocampus and is usually accompanied by spongiform degeneration in the midbrain, thalamus and striatum (for further histology details see [2] and [6]).

### Immunohistochemical detection of proteins and isoforms

**Fig 1:**
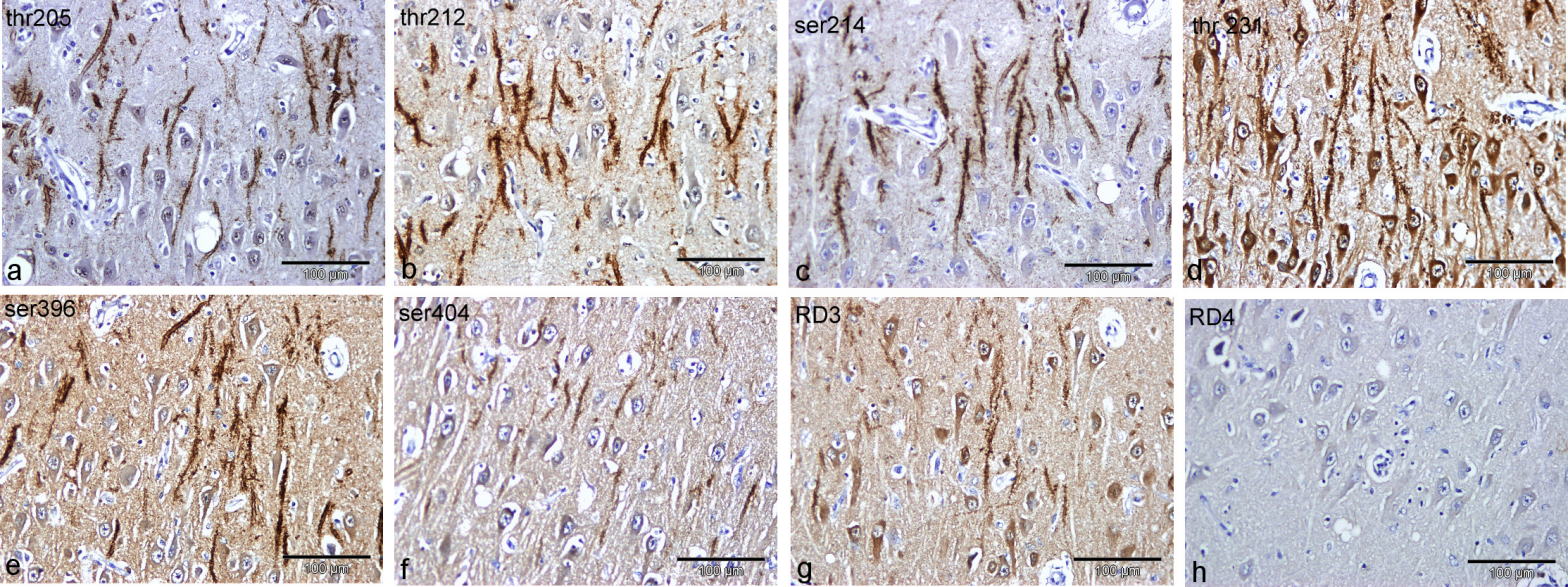
Hippocampal tau and P-tau labelling with 8 antibodies. Presence or absence of labelling of dendrites of the CA1 sector of hippocampus from a single case of IBNC following labelling with multiple tau specific antibodies. Panels show labelling with antibodies specific to hyperphosphorylated threonine or serine residues: a, threonine 205; b, threonine 212; c, serine 214; d, threonine 231; e, serine 396; f, serine 404. Labelling is also shown for antibodies specific to 3 repeat (g) or 4 repeat (h) isoforms. Bars = 100μm.

P-tau labelling was identified in all 15 IBNC brains examined and with all tau antibodies except RD4 (Fig 1A–1H). Labelling with all tau antibodies each recognising individual P-tau epitopes, albeit with different levels of sensitivity, was associated with glia, dendrites (Fig 1A–1F) and neuronal perikarya throughout the brain. P-tau immunolabelling was not present in chromatolytic neurons or degenerate axons. Where P-tau occurred, labelling of neuropil and of dendrites was also found for alpha-synuclein and ubiquitin. No labelling of IBNC brains was found for Aβ or for TDP43.

No labelling for tau or for alpha synuclein was detected in control brains of adult, aged cows lacking histological changes or from cows with neurological conditions other than IBNC. Ubiquitin labelled occasional neuronal nuclei in control cow brains but did not label dendrites, neuronal perikarya or glial cells.

### Nature and cellular association of P-tau immunolabelling

**Fig 2:**
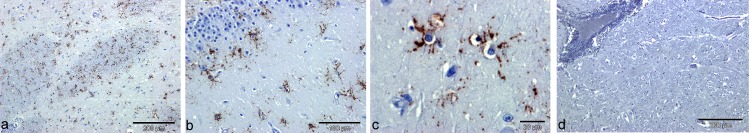
Glial P-tau labelling. Glial associated patterns of immunohistochemical labelling with AT8 antibody. (a) Striatum showing white and grey matter labelling of glial cells. (b) Detail of glial cells in hippocampus showing stellate morphology. (c) Further detail of glia labelling showing stellate pattern and loss of glial cytoplasm with maintained peripheral rim of labelling. (d) Control cow brain with encephalic listeriosis. No labelling for P-tau is present. Bars: a = 200μm; b = 100μm; c = 20μm; d = 200μm.

Glial cell—associated P-tau labelling appeared as punctuate neuropil labelling surrounding astrocytic nuclei in grey and white matter (Fig 2A). Alternately, glial cell processes were labelled to derive a stellate pattern resembling that of the tau-positive tufted astrocytes found in progressive supranuclear palsy in humans (Fig 2A and 2B). Many glial cells showed an ovoidal or circular pattern of labelling at the cytoplasmic periphery. This was seen both in combination with stellate patterns and without process labelling. Although cytoplasmic remnants were sometimes observed, most often this latter pattern was associated with dark pyknotic nuclei and loss of cytoplasm (Fig 2C).

**Fig 3:**
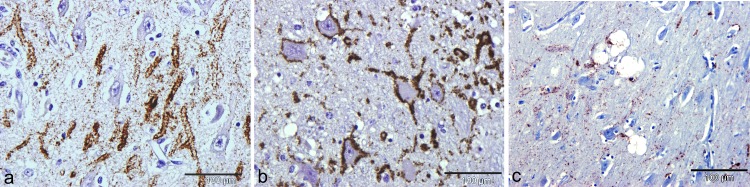
Neuronal and dendritic P-tau labelling. Dendrite and peri-neuronal tau immunohistochemical labelling patterns with AT8 antibody. (a) Detail of dendritic labelling of CA1 dendrites in hippocampus. Parallel rows of puncta suggest labelling is at or adjacent to the plasma-membrane. (b) Labelling around the perikarya and processes of neurons in the thalamus. (c) Some dendrites that are labelled with AT8 also appear to be the origins of loculated vacuoles. Bars: 100μm.

P-tau immunolabelling was found on dendrites and in a peri-neuronal pattern of labelling (Fig 3A and 3B). The labelling was in the form of a continuous puncta lining the perimeter of the dendrites, suggesting that P-tau was present on or beneath the plasmalemma (Fig 3A).

Only in a single case were intracytoplasmic neuronal inclusions of olivary neurons labelled for tau (not shown). On HE sections the P-tau positive inclusions corresponded to areas of cytoplasm with slightly refractile granules.

No labelling for P-tau of glia or dendrites was seen in control cattle brains (Fig 2D).

### Neuroanatomical patterns of labelling

The range of severity of lesions in IBNC varies considerably. In approximately half the cases lesions are confined to the neuraxis. In these cases P-tau labelling was mostly confined to glial associated patterns. The most widespread and intense P-tau accumulations were found in those cases which showed the additional lesions of hippocampal sclerosis and spongiform changes.

In cases of IBNC in which hippocampal neuronal loss were minimal or absent, glial associated tau labelling markedly predominated over dendritic and peri-neuronal patterns. Glial associated patterns of labelling occurred throughout the neuraxis, in the cerebellum, hippocampus and neocortex. In some brains with minimal histologically evident morphological lesions, glial associated labelling alone was found in hippocampus while in other brains both dendritic and glial labelling was found. Dendrite and perineuronal labelling of the hippocampus mainly involved the CA1 neuronal layer, the stratum oriens and dentate gyrus. Glial associated P-tau labelling was also present in dentate gyrus but mainly involved the stratum radiatum of the CA1 sector. In IBNC brains in which hippocampal lesions were conspicuous, glial associated tau labelling was retained throughout the neuraxis but dendrite and peri-neuronal labelling predominated in the thalamus, neocortex, and hippocampus. Where hippocampal sclerosis and atrophy occurred there was a decrease in labelling intensity corresponding to the loss of neurons and processes from the CA1 and dentate sectors. In the cerebral cortex, dendrite and perineuronal patterns of labelling were most consistently found in the entorhinal cortex but sometimes involved other cortical regions. P-tau labelling of the cerebral cortex was mainly found in the superficial and deep pyramidal neuron layers with sparing of the mid cortical layers.

Glial associated P-tau labelling was present throughout grey matter nuclei of the neuraxis, cerebellum and hippocampus. The extent of glial cell tau labelling also varied in proportion to the magnitude of hippocampal change observed in HE stained sections. The number of grey matter locations in which glial labelling was found increased in line with the severity of hippocampal changes. Sites showing glial associated labelling in minimally lesioned brains included, the cuneate nuclei, the spinal tract of the trigeminal nerve, red nucleus, substantia nigra, dorsal thalamic nuclei, hypothalamus, globus pallidus and internal capsule of the striatum. As described above glial tau labelling was also prominent in the hippocampus of minimally lesioned brains suggesting that a glial tauopathy precedes the dendritic and peri-neuronal tauopathy.

#### Relationship of P-tau to other lesions

Degenerate and chromatolytic neurons of the neuraxis were not labelled for P-tau but vacuolation in thalamus, hippocampus and cerebral cortex was related to dendritic and perineuronal patterns of labelling (Fig 3C). It is likely that P-tau accumulates on the perikarya or dendrites of vacuolated processes and neurons.

No labelling was detected with TDP-43 or with either of the Aβ peptide specific antibodies used and thus P-tau immunolablleing in IBNC cattle is not correlated with either TDP-43 or Aβ.

#### Alpha synuclein

**Fig 4:**
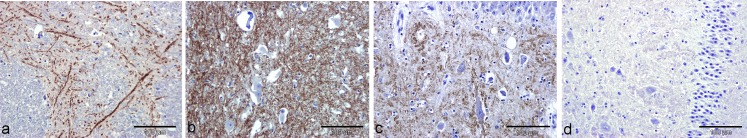
Alpha synuclein immunohistochemistry. (a) Generalised punctuate pattern of neuropil labelling in the dorso-lateral geniculate nucleus of the midbrain sparing neuronal perikarya. (b) Punctuate pattern labelling of dendrites in the substantia nigra. (c) Punctuate labelling of neuropil associated with dentate gyrus of hippocampus. (d) No labelling for alpha synuclein in the dentate gyrus of a control cow. Bars: 100μm.

Alpha synuclein labelling was also conspicuous in the hippocampus. Punctuate alpha synuclein labelling was present in neuropil surrounding neurons and their primary dendrites in the dentate gyrus (Fig 4A). Labelling predominantly occurred in a distinct zone beneath the polymorphous nuclear layer. Intracytoplasmic labelling of perivascular macrophages was also present.

At other neuroanatomic sites a pattern of parallel granular labelling occurred surrounding dendrites of the substantia nigra (Fig 4B), the globus pallidus and the entorhinal cortex where additional fine punctuate intracytoplasmic neuronal alphasynuclein labelling could also be seen. A generalised gray matter punctuate labelling was present in the dorsal lateral geniculate nucleus of the midbrain (Fig 4C) and in the grey matter of the thalamo-cortical projection fibres. These patterns of alpha synuclein accumulation resemble those occurring secondary to synaptic loss in the human brain, rather than part of a primary process of abnormal alpha synuclein accumulation.

Although amphophilic intracytoplasmic neuronal inclusion bodies are an infrequent feature of HE stained sections of IBNC we did not find evidence that these inclusions were labelled with alpha synuclein nor were typical alpha synuclein labelled Lewy bodies or neurites identified in this series.

No labelling for alpha synuclein was seen in control cattle brain tissues (Fig 4D).

#### Ubiquitin

Punctate ubiquitin labelling was present along neuronal dendrites in the dentate gyrus and in the stratum radiatum and stratum oriens of CA1 sector of the hippocampus. This pattern corresponded to the labelling found on tau positive dendrites at this site. Punctuate ubiquitin labelling was in found in association with glia of the cortical white matter, and a small proportion of dendritic processes in the deeper cortex. There was no association with vacuolation.

**Fig 5:**
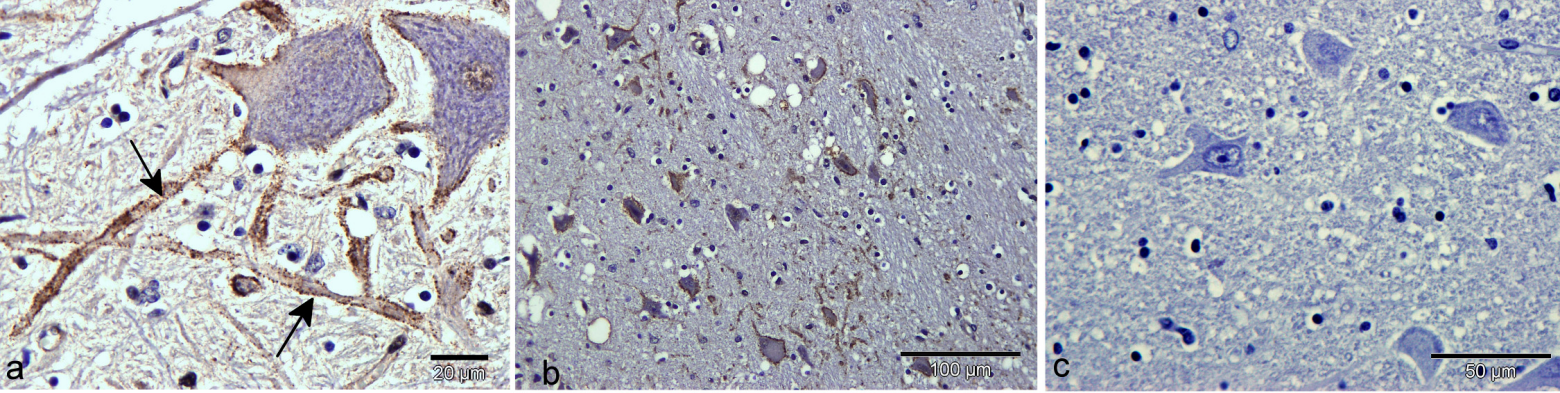
Ubiquitin immunohistochemistry. (a) Parallel punctuate labelling along dendrites (arrows) and around the perikarya of two neurons in the hypoglossal nucleus. (b) Punctuate neuronal perikaryonal land dendritic labelling in the thalamus. Note vacuolation of some affected neurons. (c) No ubiquitin labelling of neurons or neuropil in the thalamus of a control cow brain. Bars: a = 20μm; b = 100μm; c = 50 μm.

A punctuate perineuronal ubiquitin labelling pattern was conspicuous in brainstem, mid-brain and thalamus (Fig 5A and 5B) which also largely corresponded to the tau pattern at this site. Ubiquitin perineuronal labelling was found in a small number of neurons of the Dorsal Motor Nucleus of the Vagal nerve (DMNV), the reticular formation, vestibular complex, hypoglossal and red nuclei, In addition, spherical swollen axons (not shown) of the DMNV, olives and cuneate nuclei, and at several sites in the thalamus were sometimes strongly labelled for ubiquitin.

Ubiquitin labelling was not found in degenerate neuronal cytoplasm though occasional intra-nuclear inclusions were ubiquitinated.

Punctuate labelling of dendrites or perikarya was not found in control cattle tissues (Fig 5C)

#### Histology and Electron microscopy

Intra-cytoplasmic labelling suggestive of neurofibrillary tangles was not identified with Bielschowsky stain. Electron microscopy revealed abundant florid bundles of intermediate filaments that were in frequent astrocytes but clear evidence for paired and twisted filaments consistent with intra-neuronal neurofibrillary tangles were not identified.

#### Western Blotting

**Fig 6:**
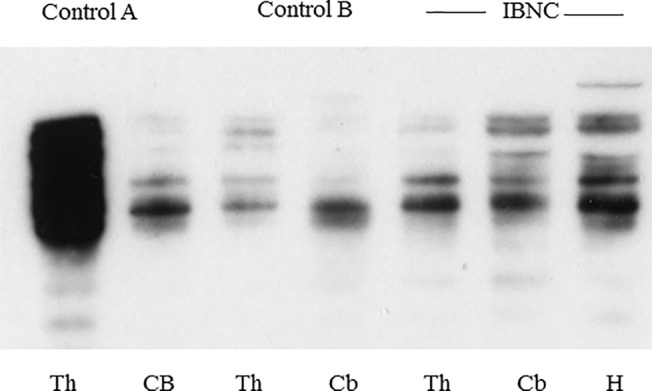
Western Blot for tau on IBNC brains and controls. Western blot analysis of tau isoforms within the thalamus (Th), cerebellum (Cb) and hippocampus (H) from a single case of IBNC. Tau isoforms in the brains of animals in which no P-tau was detected (control A and control B) following immunohistochemistry are shown on the blot for comparison.

Western blots showed the presence of multiple tau immuno-reactive bands in the brains of IBNC cattle. Similar tau signals were also obtained for controls (Fig 6).

## Discussion

Tau is phosphorylated at about 30–40 sites in normal human brains and is also minimally phosphorylated in normal adult mammalian brains. In some human disease states tau may become hyperphosphorylated with up to 60 phosphorylation sites. In animals, P- tau has been described as thread like processes in the brains of clinically normal aged animals [18] and is inconsistently associated with some experimental and naturally occurring prion diseases [19,20] as it is also in man [21]. Some presumed constitutionally expressed isoforms of tau are abundant within the neuronal cytoplasm of some animal species [16,22]. Numerous tauopathies exist in man [23]. Some tauopathies such as Primary Age Related Tauopathy (formerly known as neurofibrillary dementia) [23,24] may lack clinical features in some individuals but many tauopathies have distinctive clinico-pathological presentations [23]. In contrast, neither clinical disease nor age related syndromes have previously been associated with consistent tau hyperphosphorylation in animals. In IBNC P-tau is both invariably present and associated with neurodegenerative features [2] in cows presenting with distinct neurological signs [3]. Thus IBNC appears to be the first naturally occurring and geographically widespread tauopathy in a species other than humans.

P-tau may aggregate into intra-cellular fibrillar structures. In Alzheimer’s disease aggregated tau takes the form of paired helical filaments. These may be identified by silver impregnation methods and are usually referred to as neurofibrillary tangles. Fibrils composed of aggregated tau molecules are not confined to Alzheimer’s disease but may occur as straight or random filaments in some other human tauopathies (Table 2). IBNC appear to lack intracellular neurofibrillary tangles, as shown by negative silver stains and by electron microscopic examinations suggesting that aggregation of P-tau in IBNC may not progress to formation of filaments.

Many human neurodegenerative diseases show abnormalities of tau and microtubules and although most are considered complex proteinopathies, some familial diseases with mutations of the microtubule associated protein gene, such as familial fronto-temporal dementia are considered pure tauopathies [23,25]. No genetic information about the bovine tau gene was available for cases of IBNC, but the involvement of multiple cattle breeds and the sporadic but geographically widespread distribution of IBNC cases across Scotland [2] and elsewhere within the UK argues against a familial cause of disease.

IBNC shows hyperphosphorylation at numerous sites across the tau molecule, albeit of those sites tested some were more conspicuous than others. 3R forms of tau were recognised but not the 4R forms. Labelling of 3R tau is known to require specific pre-treatments [16] and labelling for 4R may be capricious. The absence of 4R labelling persisted when methods considered highly favourable for detection of 3 and 4R isoforms in man was used [16], but in the absence of any 4R positive bovine control tissues we should be cautious of concluding that no 4R forms were present. Some species, such as the mouse, do not generate both 3R and 4R isoforms of tau [26]. However, adult cows generate isoforms with the N-terminal extensions 3R1N, 3R2N, 4R1N, 4R2N [26], which may suggest that IBNC represents a predominantly 3R tauopathy in cattle.

Tauopathies in man are classified according to the physical state of aggregation of abnormal tau, by the molecular size and nature of P-tau, by the neuroanatomical distribution of lesions and by additional neurodegenerative features [8,23,27,28]. The Western blot data from the three IBNC brains tested suggests that tau with multiple molecular weights is present, albeit the Western blots do not test the state of phosphorylation of the tau recognised. The immunoblot data therefore confirms the presence of multiple tau molecular states in IBNC brains, but the similar presence of tau in control adult cattle brains makes further interpretation difficult. Significant additional work to test specifically for the presence of hyperphosphorylated tau forms will be necessary to provide meaningful interpretation. Nevertheless both the immunoblotting and immunohistochemical data are consistent with IBNC brains containing multiple molecular weight isoforms of P-tau, potentially with a range similar to that found in Alzheimer’s disease. However, plaques or diffuse accumulations of Aβ_1–40_ or Aβ_1–42_ were absent from IBNC brains. Hippocampal sclerosis in man may be associated with TDP-43 immunolabelling, but this too was absent in IBNC. The predominant glial tau association of IBNC has similarities to the tau gliopathy reported for argyrophilic grain disease, progressive supranuclear palsy and corticobasal degeneration [22] but, in contrast to the predominant 3R tauopathy found in IBNC, these human neurodegenerations are all 4R tauopathies (Table 2). In common with other protein misfolding diseases, IBNC appears to be a complex proteinopathy with evidence for additional secondary accumulation of alpha synuclein, ubiquitin, and possibly PrP [5]. However there is no evidence of Lewy bodies, neurites or other primary alpha synuclein pathology such as is found in Parkinson’s disease. Thus the nature of the tauopathy in IBNC does not precisely parallel that of any of the existing human tauopathies ([22] and Table 2); however, it appears to represent a naturally-occurring predominantly 3R tauopathy in a non-primate species.

IBNC is a chronic progressive neurological illness that is widespread and occurs sporadically throughout the UK in aged cattle. Clinical signs of IBNC include behavioural changes, increased apprehension and locomotor problems such as tremor and stiffness of gait, and weight loss [2,3]. Difficulty in swallowing with increased salivation is also reported is some cases. Attempts to find the cause of IBNC have so far been unsuccessful. Small scale and largely unpublished studies of IBNC have not identified infectious, metabolic or micronutrient deficiencies that are known to cause neurodegeneration in cattle [6]. It is now widely understood that both genetic and environmental factors contribute to the pathogenesis of some human dementias and several environmental risk factors have been identified for some human Parkinsonian syndromes. In particular exposure to insecticides and herbicides and employment in the agricultural or horticultural industries increases risk [29,30,31]. Although the mechanisms remain uncertain, it has been suggested that diverse toxins may interact with alpha synuclein to initiate aggregation of alpha synuclein in Parkinson’s disease [32]. Experimental exposure of rodents to the insecticides rotenone, [33] the herbicide paraquat or the fungicide maneb (a dithiocarbamate) each results in striato-nigral dopaminergic neuron loss [32]. In the absence of any specific aetiological cause of IBNC yet identified, and based on the existing epidemiology and presence of P-tau we have considered the possibility that IBNC might also have a significant environmental component. Epidemiological investigations to explore usage of herbicides, insecticides, parasiticides and other chemicals used in agriculture would be helpful in future investigations of IBNC and might provide further insight into some causes of neurodegeneration in man.
